# Enterocutaneous Fistula Caused by an Ingested Chicken Bone in an Adult: A Case Report

**DOI:** 10.1155/cris/7208736

**Published:** 2026-01-28

**Authors:** T. B. Sørensen, M. W. Ørntoft, C. Jaensch

**Affiliations:** ^1^ Surgical Department, Gødstrup Hospital, Herning, Denmark; ^2^ Department of Clinical Medicine, Aarhus University, Aarhus, Denmark, au.dk; ^3^ Surgical Research Unit, Gødstrup Hospital, Herning, Denmark; ^4^ NIDO | Centre for Research and Education, Gødstrup Hospital, Herning, Denmark

**Keywords:** case report, conservative management, enterocutaneous fistula, foreign body ingestion, surgical management

## Abstract

**Introduction and Importance:**

Enterocutaneous fistulas (ECFs) caused by ingested foreign bodies are extremely rare. Accurate fistula localization is essential for guiding management, especially in comorbid patients at high surgical risk.

**Case Presentation:**

We report a 76‐year‐old woman who developed an ECF secondary to a swallowed chicken bone. Initial imaging suggesting a high small‐bowel fistula and severe peristomal skin breakdown prompted consideration of surgery. Extended conservative management allowed skin healing, and follow‐up imaging revealed a more distal fistula, supporting continuation of nonsurgical management.

**Clinical Discussion:**

Management of ECF is challenging due to morbidity from intestinal failure, electrolyte disturbances, sepsis, and skin damage. Optimal care requires multidisciplinary strategies guided by sepsis control, nutritional support, anatomical assessment, and procedure planning (SNAP framework).

**Conclusion:**

This case highlights the importance of individualized management and careful assessment of fistula location. It also illustrates that prolonged conservative management can achieve acceptable outcomes in high‐risk patients.

## 1. Introduction

An enterocutaneous fistula (ECF) represents an abnormal communication between the gastrointestinal tract and the skin. The majority of ECFs develop as postoperative complications [1] although inflammatory bowel disease (IBD), trauma, malignancy, and radiation are also recognized etiologies. Ingested foreign bodies are a very uncommon cause of ECF, with only a handful of cases reported in the literature [2–6]. The true incidence is naturally higher, but the very few reported cases underline the rarity of this phenomenon. Ingested foreign bodies more frequently result in gastrointestinal perforation, bleeding, or abscess formation [7]. In addition, a number of iatrogenic ECFs have been described following migration of medical materials, most often surgical meshes.

Here, we present a rare case of an ECF caused by a swallowed chicken bone in a patient with a large ventral hernia. This case highlights the importance of accurate fistula localization for guiding management, especially in comorbid patients where surgery carries high risk, and demonstrates that prolonged conservative management can achieve favorable outcomes.

## 2. Case Description

### 2.1. Patient Background

A 76‐year‐old woman with relevant comorbidities, including type 2 diabetes and prior breast cancer, presented to the emergency department with a new abdominal wound. Seven years earlier, the patient had undergone an open Hartmann’s procedure with colostomy for perforated diverticulitis, complicated by intestinal perforation, fascial dehiscence, and vacuum‐assisted closure, leaving her with a large ventral hernia.

### 2.2. Presentation and Diagnosis

The patient awoke during the night with acute pain and bleeding from her hernia. Examination revealed a foreign body protruding from a central wound (Figure 1A). CT demonstrated an elongated object within the subcutaneous tissue, adjacent to small bowel loops, without abscess, free fluid, or pneumoperitoneum (Figure 1C,D). At the bedside, the foreign body was removed and identified as a chicken bone (Figure 1B). Enteric fluid drained from the wound, consistent with an ECF. A subsequent CT with oral contrast suggested an origin near the jejunoileal junction (Figure 2A).

Figure 1(A) Abdominal wound at presentation with a foreign body protruding through the skin. (B) Extracted chicken bone. (C) Sagittal and (D) axial abdominal CT scans showing the foreign body (arrows) within the subcutaneous tissue adjacent to small bowel loops.

**Figure:**
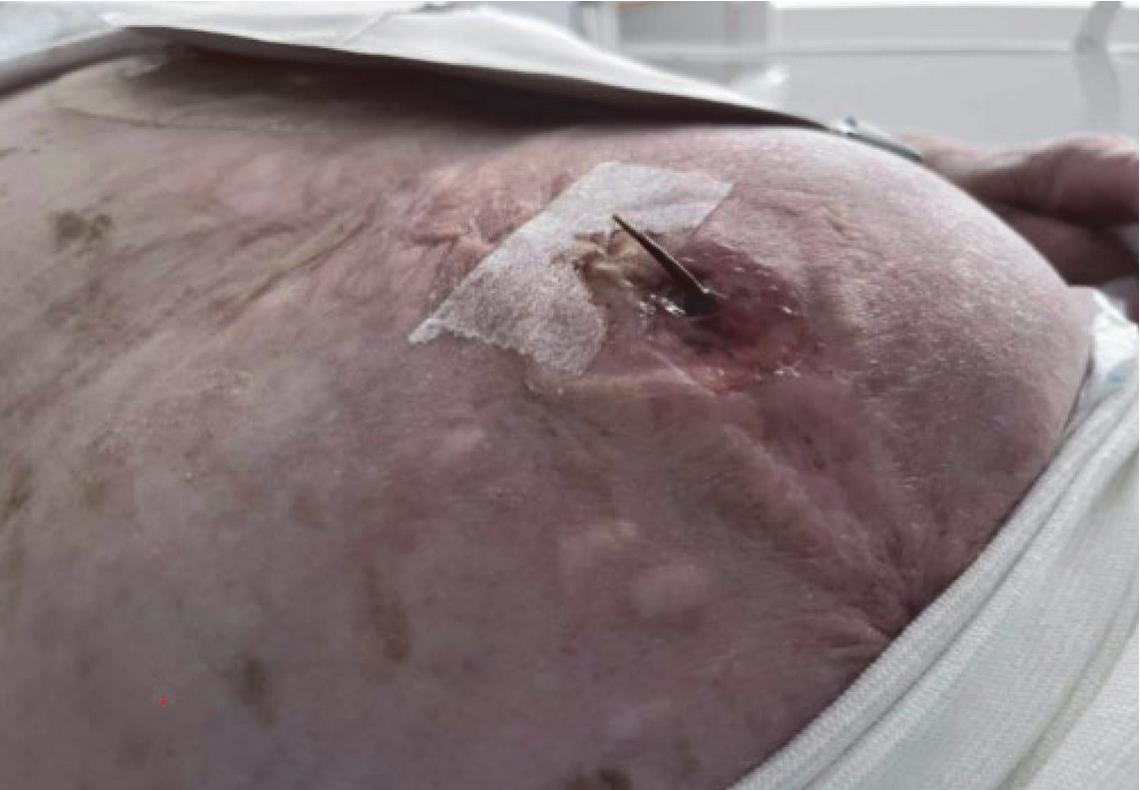
(A)

**Figure:**
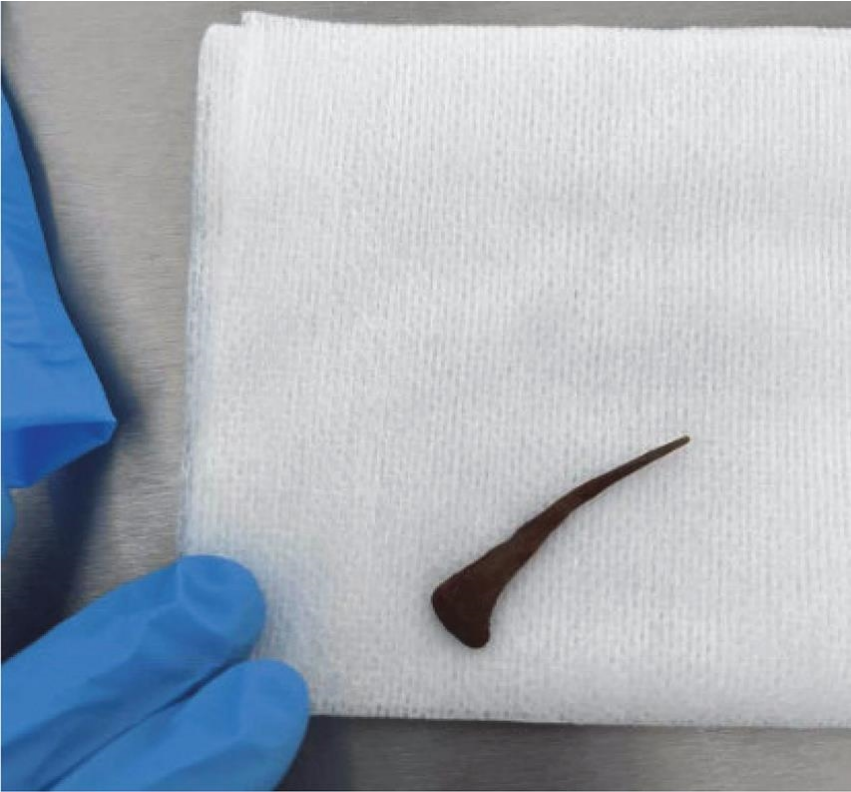
(B)

**Figure:**
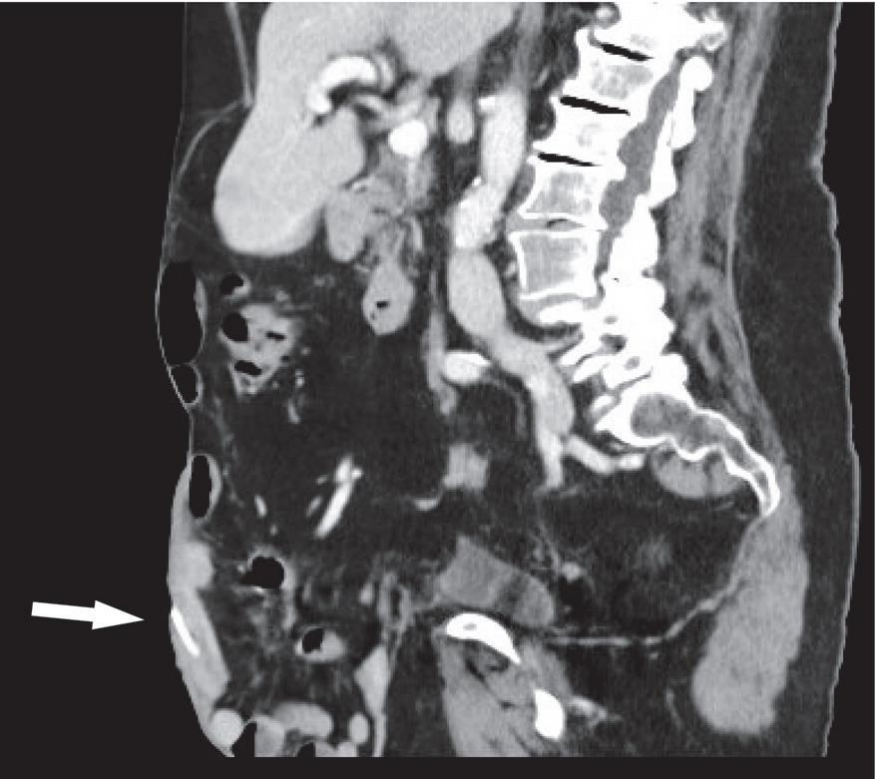
(C)

**Figure:**
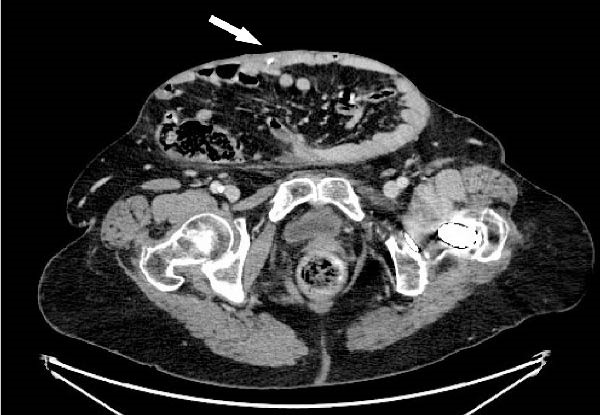
(D)

Figure 2(A) Initial abdominal CT with oral contrast showing the fistula opening (arrow) and a contrast‐filled pouch (arrowhead). The fistula tract itself is not visible in this frame. (B) Sagittal small bowel follow‐through X‐ray demonstrating a more distal fistula origin, with contrast leakage through the tract (arrow).

**Figure:**
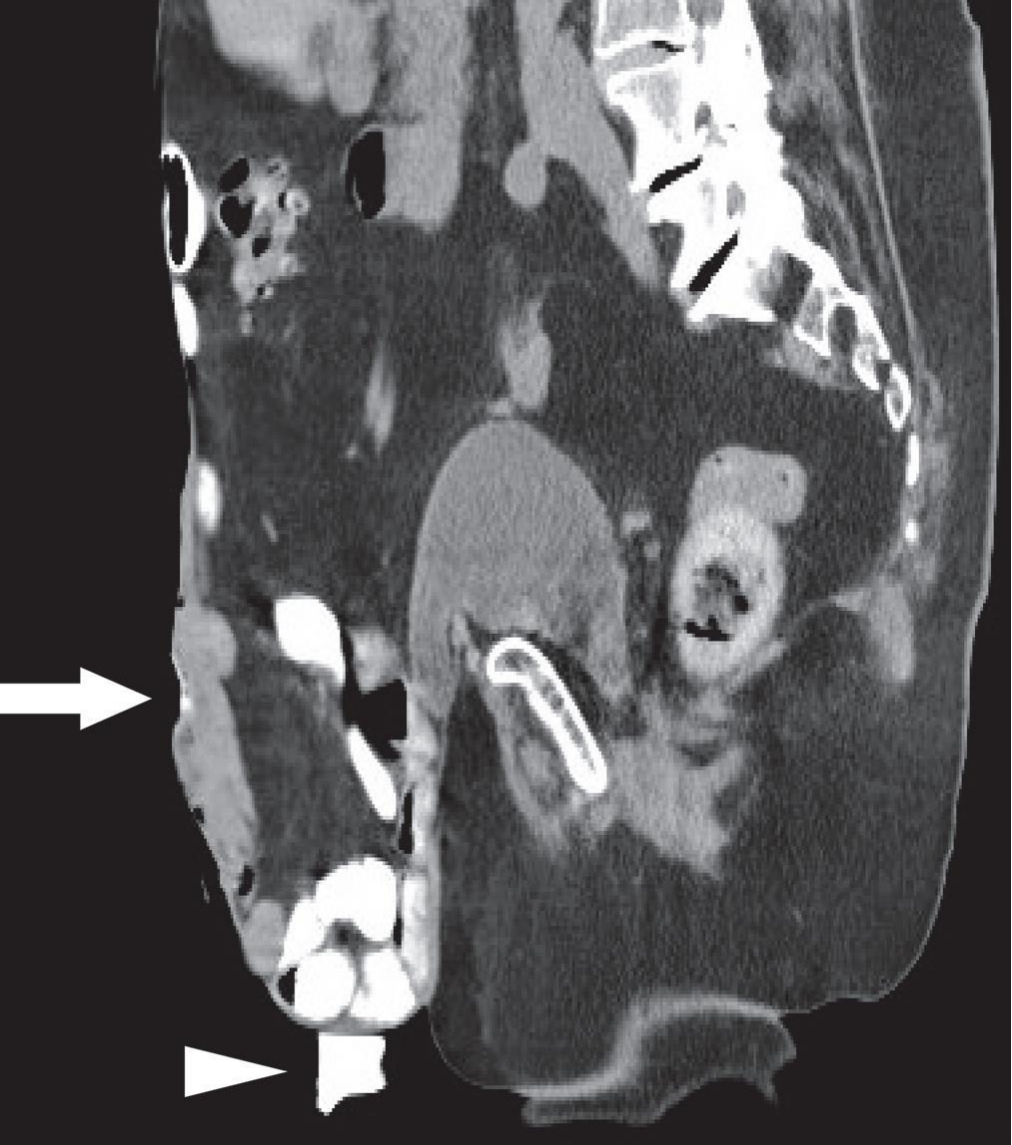
(A)

**Figure:**
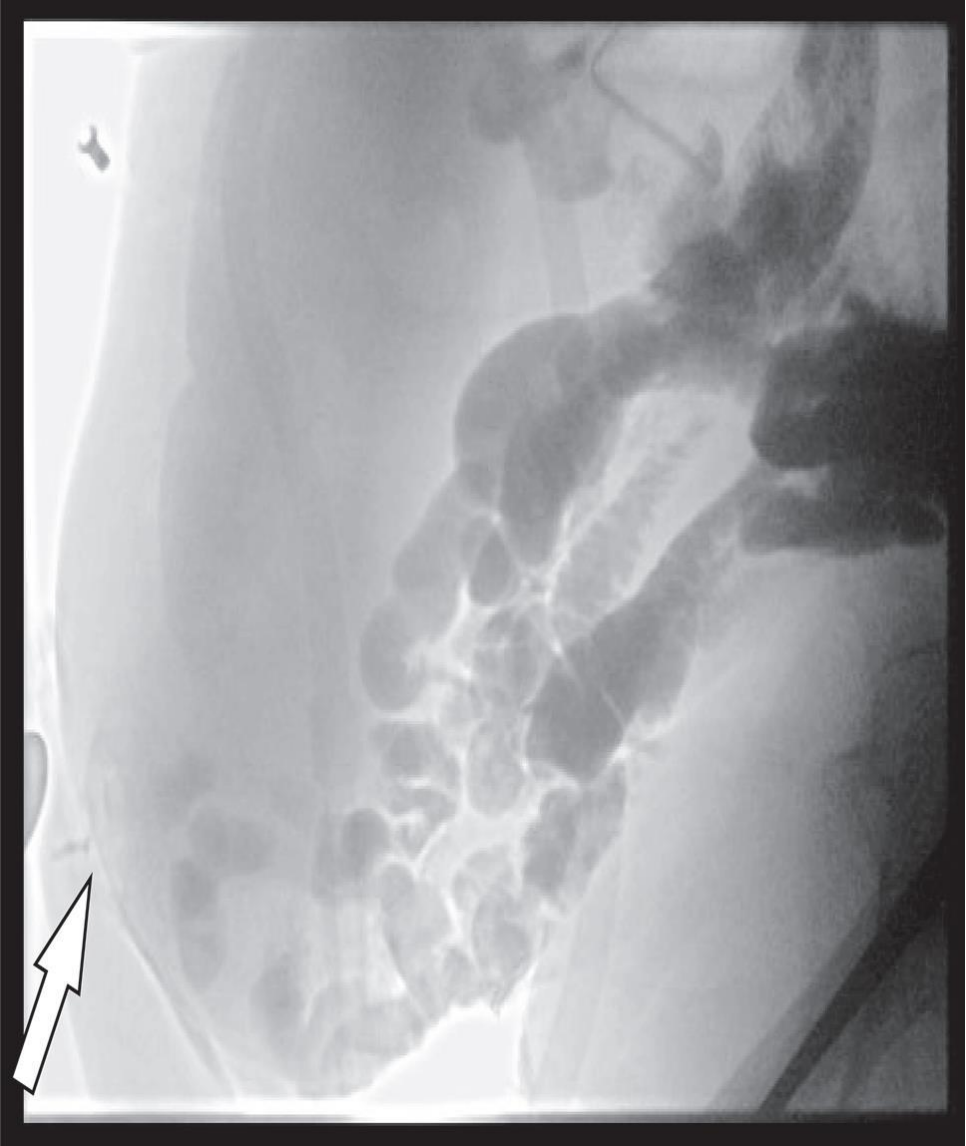
(B)

### 2.3. Treatment

Conservative outpatient management was initially pursued, anticipating spontaneous closure. After 5 weeks, the fistula remained patent with moderate output and peristomal skin breakdown (Figure 3A). Despite high‐risk anatomy, poor healing potential, and comorbidities, surgical management was decided upon. Following discussion with a tertiary hernia center, an explorative laparotomy with fistula closure—without abdominal wall reconstruction—was planned. Yet before surgery could be undertaken, the patient was readmitted with dehydration and progressive peristomal skin breakdown (Figure 3B), prompting initiation of TPN and placement of a Foley catheter to divert effluent enteral fluid.

Figure 3(A) Five weeks after initial admission: patent fistula with peristomal skin breakdown. (B) Six weeks after admission: readmission with dehydration and worsening peristomal skin breakdown. (C) Three months after admission: wound improvement around the fistula. (D) Three and a half months after admission: near‐complete peristomal skin healing; the fistula has widened, functioning as a natural small‐bowel stoma.

**Figure:**
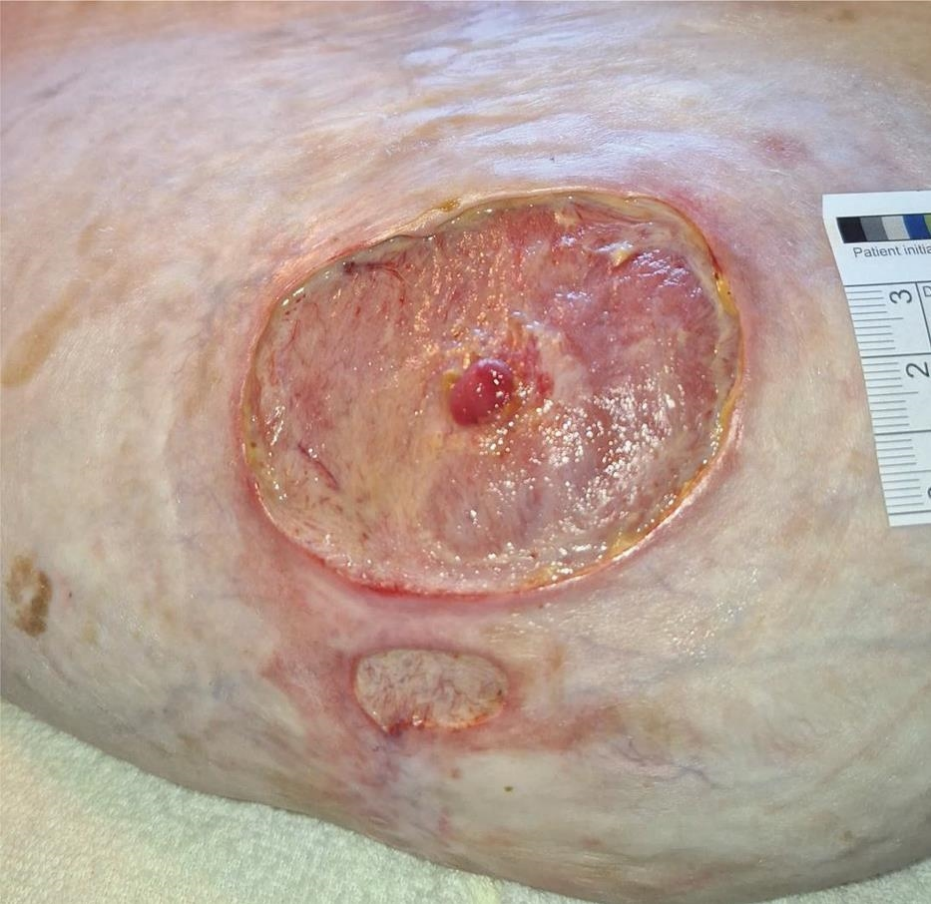
(A)

**Figure:**
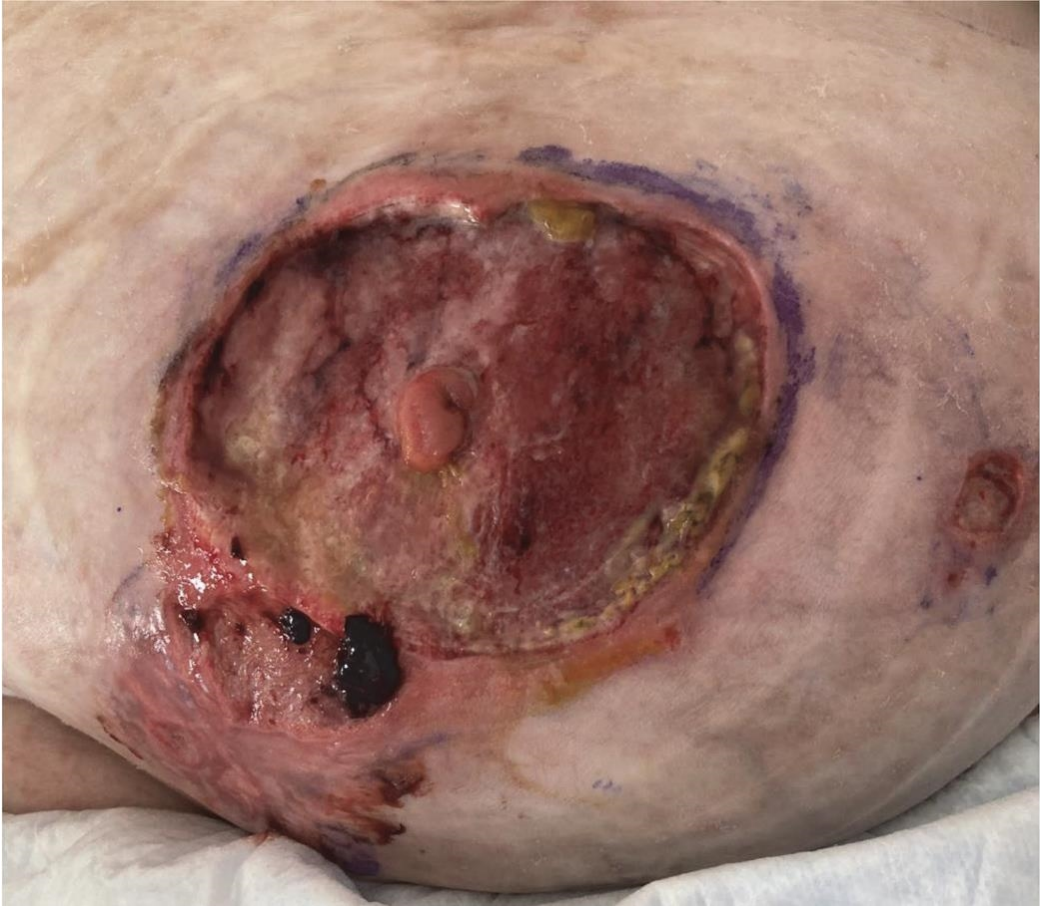
(B)

**Figure:**
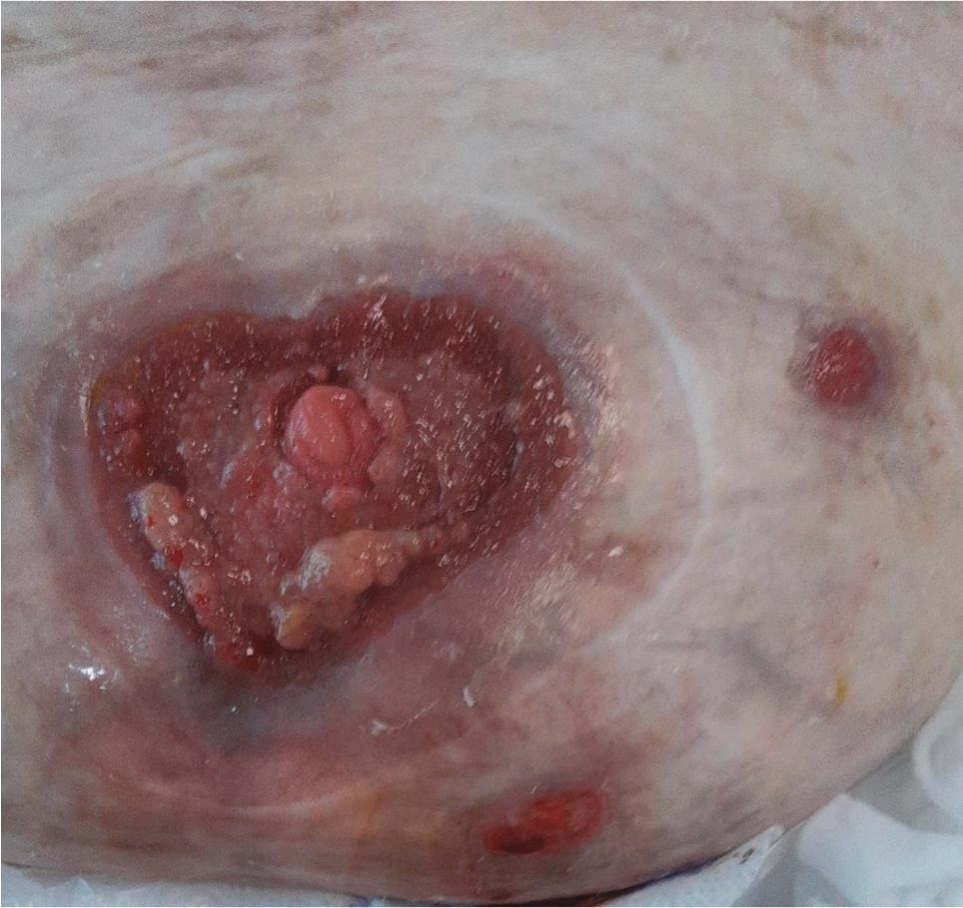
(C)

**Figure:**
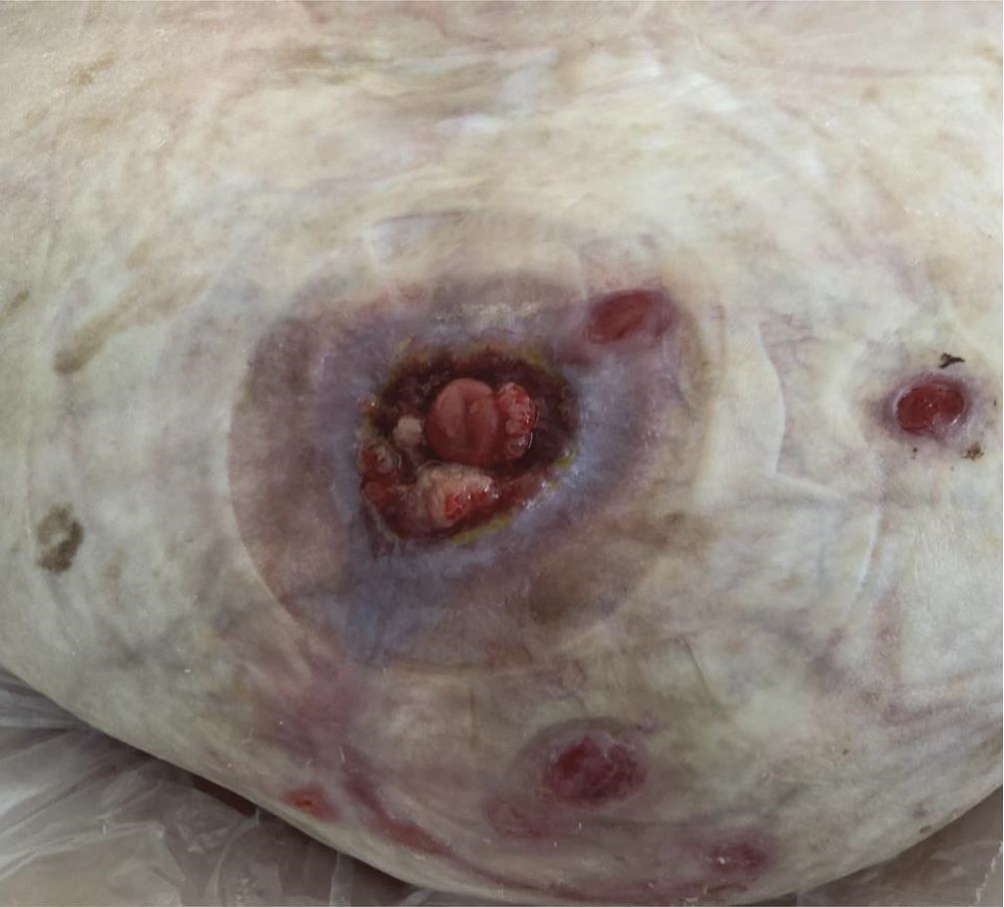
(D)

During this admission, the patient developed line‐associated endocarditis, requiring 6 weeks of intravenous antibiotics. This delay allowed the peristomal wound to improve (Figure 3C,D) and oral intake to resume. A small bowel follow‐through demonstrated a fistula origin in the ileum, more distal than initially assumed, with a clinically significant length of functioning small bowel proximal to the fistula (Figure 2B), leading to continuation of conservative management.

### 2.4. Outcome

After 3 months of hospitalization, the patient was discharged to a rehabilitation facility. At discharge, her stoma output was variable, averaging ~1000 mL/day. She was able to meet her nutritional needs through oral intake, including electrolyte‐containing drinks. A dedicated stoma appliance covering the fistula allowed most of the effluent to be contained without leakage or recurrent skin breakdown. The patient was discharged with close follow‐up by stoma nurses and planned outpatient visits with her physician. Given the patient’s significant comorbidities and high surgical risk, this high‐output fistula was considered an acceptable outcome at discharge.

## 3. Discussion

Management of ECF remains one of the most challenging aspects of colorectal surgery, associated with significant morbidity and mortality due to intestinal failure, electrolyte disturbances, sepsis, and peristomal skin damage [8, 9]. Optimal outcomes require a multidisciplinary approach addressing fluid/electrolyte management, nutrition, infection control, wound care, and appropriately timed surgical intervention [10]. These principles are summarized in the “SNAP” framework: sepsis (and skin care), nutrition, anatomy (location of fistula), and procedure planning [11].

This case illustrates a rare etiology of ECF caused by an ingested foreign body and highlights challenges across all aspects of the “SNAP” framework.

Initially, a conservative strategy was pursued, anticipating spontaneous closure. A recent systematic review and meta‐analysis of 2806 patients reported that 44.8% of patients undergoing conservative management achieved spontaneous closure [1]. Closure is less likely in patients with high output, long tracts, malignancy, active inflammation, or poor nutritional status [12]. Among the patients who underwent surgery, 86% achieved successful closure, although some required more than one operation. Recurrence after any type of treatment was reported in 15.1% of patients, and overall mortality approached 9.6% [1]. These figures are derived from heterogeneous studies, so generalizability should be interpreted with caution.

Accurate anatomical localization is crucial in ECF management. In this case, the initial CT scan suggested a high small‐bowel origin, prompting consideration of high‐risk surgery. A subsequent small bowel follow‐through revealed a more distal fistula, allowing continuation of conservative management and avoidance of unnecessary high‐risk surgery in this comorbid patient. Notably, the delay caused by treatment of line‐associated endocarditis proved fortuitous, allowing peristomal skin healing and further anatomical clarification, which ultimately supported continued conservative management.

## 4. Conclusion

Foreign body ingestion is a very uncommon cause of ECF, and this case highlights the importance of individualized, multidisciplinary management. It illustrates both the rarity and complexity of ECF management, emphasizing how careful assessment of sepsis, nutrition, precise fistula localization, and timing can guide conservative versus surgical strategies.

This case also demonstrates that, in a comorbid patient where surgery carries high risk, careful monitoring and prolonged conservative management can allow skin healing and achieve an acceptable clinical outcome.

**Nomenclature**
ECF: Enterocutaneous fistula
CT: Computed tomography
IBD: Inflammatory bowel disease
SNAP: Sepsis (and skin care), nutrition, anatomy, procedure planning
TPN: Total parenteral nutrition.

## Author Contributions

Study concept or design: C. Jaensch. Data collection: T. B. Sørensen. Data analysis or interpretation: T. B. Sørensen. Writing – original draft: T. B. Sørensen. Writing – review and editing: T. B. Sørensen, C. Jaensch and M. W. Ørntoft. Guarantor: C. Jaensch.

## Funding

No funding was provided for the completion of this manuscript.

## Ethics Statement

Ethical approval is not required from the Danish Council of Ethics for publication of anonymized case reports regarding treatment with nonexperimental treatment.

## Consent

Written informed consent was obtained from the patient for publication of this case report and accompanying images. A copy of the written consent is available for review by the Editor‐in‐Chief of this journal on request.

## Conflicts of Interest

The authors declare no conflicts of interest.

## Data Availability

Data sharing is not applicable to this article as no datasets were generated or analyzed during the current study.
